# Biomimetic Silica Nanoparticles Prepared by a Combination of Solid-Phase Imprinting and Ostwald Ripening

**DOI:** 10.1038/s41598-017-12007-0

**Published:** 2017-09-14

**Authors:** Elena Piletska, Heersh Yawer, Francesco Canfarotta, Ewa Moczko, Katarzyna Smolinska-Kempisty, Stanislav S. Piletsky, Antonio Guerreiro, Michael J. Whitcombe, Sergey A. Piletsky

**Affiliations:** 1 Department of Chemistry, College of Science and Engineering, University of Leicester, Leicester, LE1 7RH UK; 2 MIP Diagnostics Ltd., Fielding Johnson Building, University of Leicester, Leicester, LE1 7RH UK; 3 Present Address: Universidad Católica de la Santísima Concepción, Facultad de Ciencias, Departamento de Química Ambiental, Alonso de Ribera, 2850 Concepción, Chile

## Abstract

Herein we describe the preparation of molecularly imprinted silica nanoparticles by Ostwald ripening in the presence of molecular templates immobilised on glass beads (the solid-phase). To achieve this, a seed material (12 nm diameter silica nanoparticles) was incubated in phosphate buffer in the presence of the solid-phase. Phosphate ions act as a catalyst in the ripening process which is driven by differences in surface energy between particles of different size, leading to the preferential growth of larger particles. Material deposited in the vicinity of template molecules results in the formation of sol-gel molecular imprints after around 2 hours. Selective washing and elution allows the higher affinity nanoparticles to be isolated. Unlike other strategies commonly used to prepare imprinted silica nanoparticles this approach is extremely simple in nature and can be performed under physiological conditions, making it suitable for imprinting whole proteins and other biomacromolecules in their native conformations. We have demonstrated the generic nature of this method by preparing imprinted silica nanoparticles against targets of varying molecular mass (melamine, vancomycin and trypsin). Binding to the imprinted particles was demonstrated in an immunoassay (ELISA) format in buffer and complex media (milk or blood plasma) with sub-nM detection ability.

## Introduction

Molecularly imprinted materials are prepared in the presence of a template species in order to create a site (or cavity) in the material complementary in size, shape and electrostatic environment to the template. Removal of the template reveals the imprints which are capable of selective and specific recognition of the target molecules^1–3^. A variety of materials are capable of being imprinted; most commonly organic polymers. Inorganic materials however have a much longer history in molecular imprinting and the formation of specific recognition sites in silica was noted as far back as the 1930 s and 40 s^4, 5^.

We recently introduced a new method for the synthesis of molecularly imprinted polymers based on a solid-phase synthesis approach^6–10^. This method results in the preparation of high-affinity molecularly imprinted nanoparticles (nanoMIPs) formed at the solid-liquid interface at an immobilised template fixed to the solid surface. Selective washing and elution steps ensure that the nanoMIPs are subjected to an affinity purification step during their synthesis. The particles are soluble, imprinted nanoscale objects resembling antibodies in their recognition properties, but with the stability and robustness of synthetic polymers. An important distinction can be made concerning these nanoMIPs when comparing them to traditionally prepared imprinted polymers in that, in the solid-phase approach, the MIPs are removed from the template, rather than the template being removed from the MIP. This means that the nanoMIPs are template-free and the template (still attached to the solid phase) may be re-used. The antibody-like properties of nanoMIPs prepared by the solid-phase approach have been demonstrated by their use in the immunoassay (ELISA) format^11–13^ and in sensors^14–17^ for the sensitive detection of a range of analytes, from small molecules to proteins. It was therefore of interest to us to investigate whether solid-phase imprinting could be applied to other classes of imprintable materials, in particular silica. In order to prepare silica in the presence of immobilised templates we compared methods of sol-gel particle synthesis. The standard methods involve hydrolysis of small molecule precursors, such as tetraethoxysilane, follow by a polycondensation phase. This however requires aqueous acid or base to initiate the process^18^, making it unsuitable for general use with peptides, proteins and other biomolecular targets. Other methods, using for example acetic anhydride^19^, may also result in unwanted side reactions with biomolecules. In general much milder reaction conditions should be used, therefore we decided to explore the phenomenon of Oswald ripening as a new method for the preparation of molecularly imprinted silica nanoparticles in combination with the solid-phase approach.

## Results and Discussion

### Ostwald Ripening

Ostwald ripening (OsR) is the physical phenomenon by which large crystals or particles grow from those of smaller size due to differential solubility related to differences in surface energy^20^. Ostwald ripening is practically always present when nanoparticles (either organic or inorganic) are dispersed in solution. This phenomenon is observed with a variety of materials, including polymers, metals and inorganic oxides such as SiO_2_, SnO_2_ and TiO_2_^21, 22^. Ostwald ripening of silica originates from surface instability of silicon dioxide and is driven by differences in chemical potential between particles of different size and shape. The local radius of curvature and ratio of surface area to volume accounts for the particle’s surface energy, which is greatest in the case of small particles or those with rough surfaces. Under kinetically favourable conditions these high surface energy particles dissolve preferentially, with the material being deposited onto the larger particles. In silica the dissolution proceeds via cleavage of siloxane bridges at the surface, resulting in the release of soluble silicic acid^23, 24^. We therefore set out to investigate the feasibility of introducing specific binding sites in commercial silica nanoparticles by molecular imprinting, facilitated by the OsR process.

**Figure 1:**
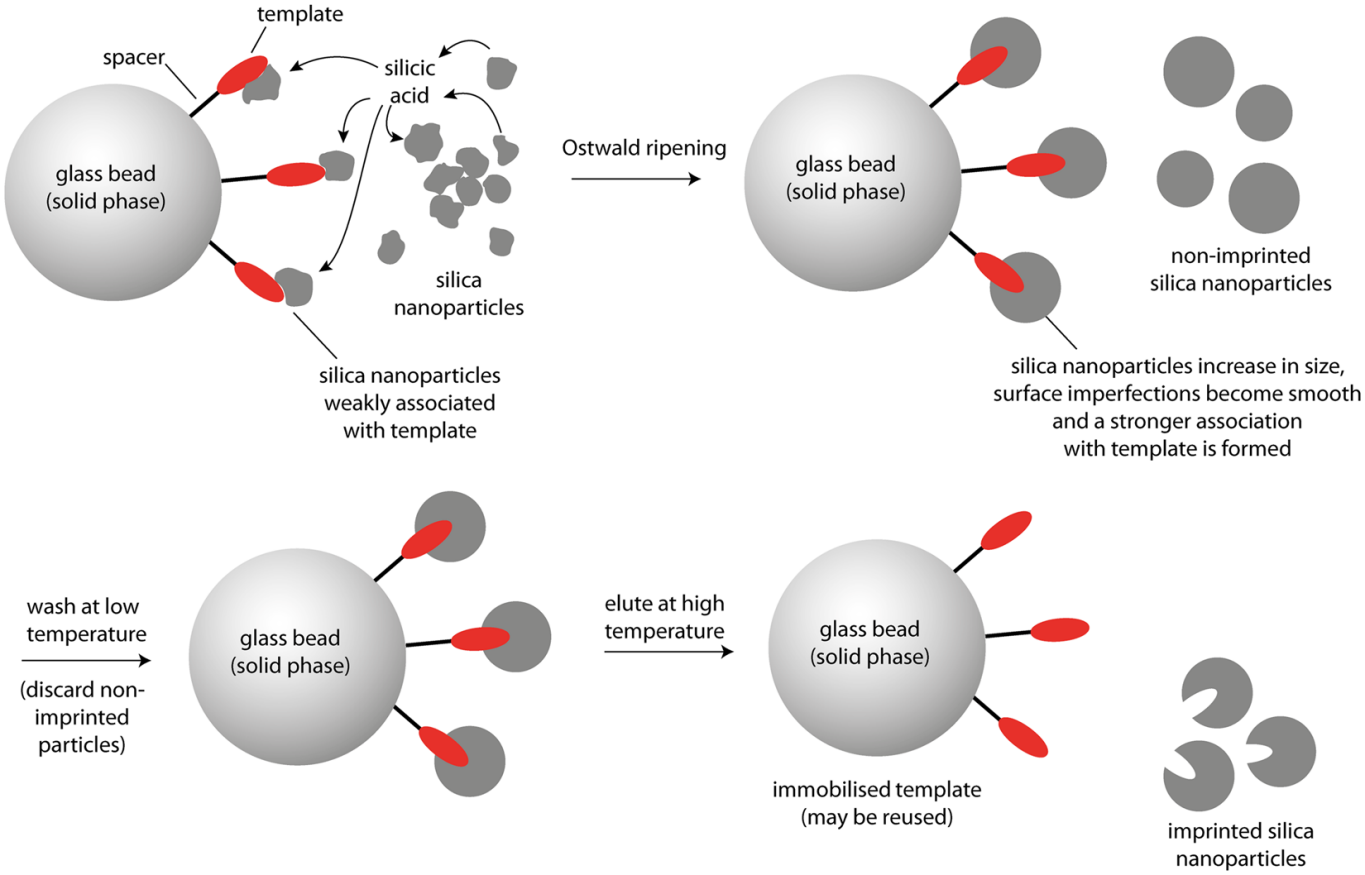
Schematic representation of the preparation of surface molecularly imprinted silica nanoparticles by the process of Ostwald ripening in the presence of an immobilised template.

To this end, the ripening process was performed by incubation of 12 nm silica nanoparticles suspended in phosphate buffer in the presence of several template molecules, immobilised on solid supports. As mentioned above, OsR of silica nanoparticles proceeds due to differences in surface energy; silica at highly curved surfaces will dissolve at an enhanced rate and re-condense preferentially in crevices and other surface defects, resulting in smoothing of the particle surface^25^. The situation is somewhat different for immobilised particles or particles in contact with each other. Since solubility is extremely low at sites with negative radii of curvature, i.e., at regions of particle-particle or particle-surface contact, dissolved silicic acid will tend to condense in these areas. A schematic of the proposed application of OsR in the preparation of molecularly imprinted silica nanoparticles in the presence of a solid support is shown in Fig. 1. It is hypothesised that some nanoparticles will initially form non-specific complexes with immobilised template molecules through electrostatic and hydrophobic interactions. The soluble intermediate, silicic acid, will condense around areas of interaction between nanoparticles and the immobilised template, turning these areas of non-specific interaction into specific binding sites. This is a generic process which should, in theory, allow a range of different types of templates to be imprinted in silica nanoparticles, under milder conditions than alternative sol-gel methods.

The kinetics of the process depends on many factors including pH, ionic strength of the solution, temperature, size and concentration of silica particles^26, 27^. The ripening process can be accelerated by addition of phosphate, borate and fluoride ions^23, 24, 28^. However, in the present application, phosphate ions were selected as more convenient catalysts for OsR, due to practical and environmental reasons. To demonstrate the generic nature of this imprinting method, we prepared imprinted silica nanoparticles against low and medium molecular weight templates (melamine, vancomycin) and also against one whole protein, trypsin.

### Synthesis of Imprinted Silica Nanoparticles

**Figure 2:**
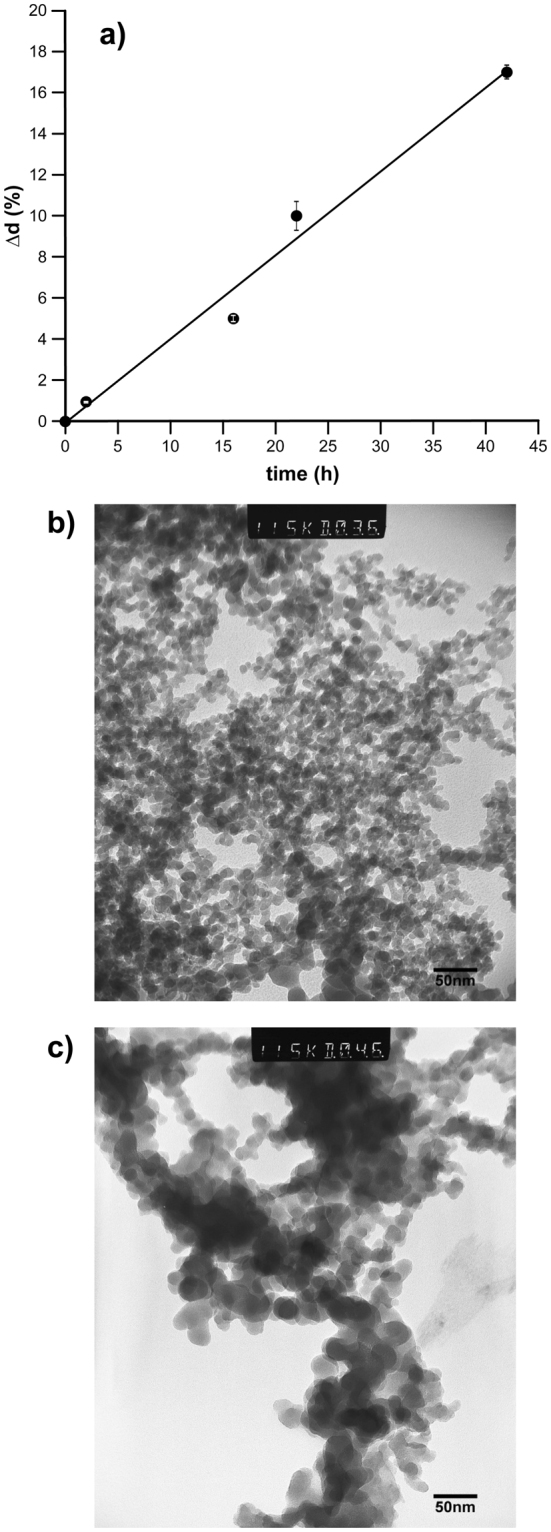
(**a**) Change in diameter of 12 nm silica nanoparticles incubated in 50 mM phosphate buffer, (pH 7.2) with time, according to dynamic light scattering (DLS) measurements. TEM images of silica nanoparticles (**b**) before and (**c**) after solid-phase imprinting of melamine by exposure to phosphate buffer for 2 hours.

It is essential that the imprinting process occurs under controlled, preferably physiological, conditions within a reasonable period of time. This will ensure that the native conformation of biological templates, such as proteins, will be preserved. For this reason we have chosen 50 mM phosphate buffer (pH 7.2) as catalyst for the OsR-based imprinting process. The amount of silica used during imprinting (1 mg mL^−1^ buffer) was selected, taking into account the stability of the colloidal dispersion, where higher concentrations would lead to precipitation over time. Evolution of the size of silica nanoparticles suspended in phosphate buffer was monitored by dynamic light scattering (DLS), Fig. 2. During OsR of silica particles in solution and in the absence of template or solid-phase, the recorded average rate of increase in diameter was 0.4% h^−1^. For successful imprinting, the incubation time should allow the template features to be reproduced on the nanoparticle surface. Since most proteins are under 5 nm in diameter, 2–6 h of OsR was therefore initially considered as an appropriate starting point for surface-imprinting of both small molecules and proteins, whilst preventing permanent occlusion of the smaller templates in the silica layer. The ripening process was then repeated, but this time in the presence of immobilised melamine at the surface of glass beads, which were exposed to silica nanoparticles in phosphate buffer for 2, 4 and 6 hours.

After ripening, the solid-phase was washed with water and the retained imprinted material eluted by raising the temperature of the solid-phase to 70 °C (see Experimental Section for full details). After 2 hours of imprinting only larger particles of ~20 nm diameter were collected during the elution step (Fig. 2c). Non-imprinted particles were removed during the cold washing steps. Particles exposed to the same conditions for longer periods grew to even greater size (40–60 nm in 4–6 h). The process was accompanied by the formation of irregular lumps of sol-gel mixed with the nanoparticles. This, together with the fact that such large particles might fully entrap some templates and their excessive size may limit their usefulness in assays, lead us to select 2 h as the optimal ripening/imprinting time. However, these results were inconsistent with those obtained in solution, where the process was much slower. This could possibly have been due to an accelerated ripening process close to the surface of the solid-phase, together with the preferential collection of only larger nanoparticles (where imprinting occurs) after the washing steps. Nanoparticles were also imprinted against vancomycin and trypsin, following the same conditions seen to be optimal for melamine. The yield of the process, when using 60 g of template-derivatised solid-phase and 50 mg silica was 12.8, 9.6 and 5.6 mg of imprinted silica respectively, for melamine, vancomycin and trypsin (12–25% yield on average). The yield is limited by the availability of the template species which was previously estimated to be present at a density of between 0.01 to 0.26 molecules per nm^2^ on the glass surface^6^. The stability of imprinted nanoparticles was assessed under different conditions. When stored dry, nanoparticles imprinted with melamine kept their recognition properties for at least two months (assessed by ELISA-type assays). Silica is unstable under conditions of high pH and especially when in the presence of phosphate ions. Incubation of melamine-imprinted silica nanoparticles in phosphate buffer, pH 8.5, for one hour resulted in complete loss of recognition properties (erasure of memory), again assessed by ELISA-type assays (results not shown). Clearly the mobility of silicic acid under these conditions is too high to preserve the binding sites. For this reason, imprinted silica nanoparticles should be stored under dry conditions or, if in aqueous dispersion, at slightly acidic pH. Accordingly, the use of these particles under aqueous conditions should also be limited only to acidic or neutral pH.

### Recognition Properties of Imprinted Silica in the Immunoassay Format

**Figure 3:**
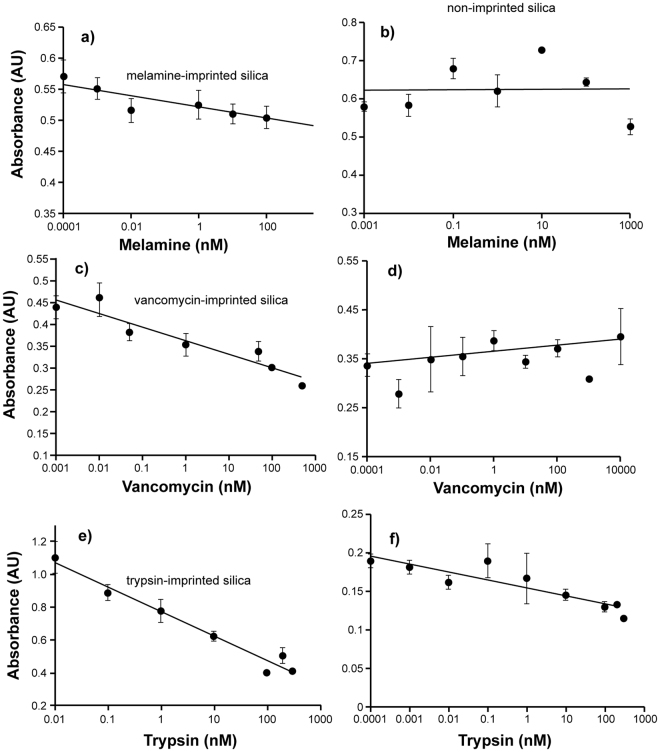
Competitive binding between free analyte and the corresponding HRP conjugates in competitive ELISA format using immobilized silica NPs in 96 well polystyrene microplates. In (**a**,**c** and **e**) only the linear ranges are depicted, no response was observed for lower analyte concentrations whilst saturation is observed at higher concentrations. (**a**) melamine with melamine-imprinted silica NPs; (**b**) melamine with non-imprinted silica NPs; (**c**) vancomycin with vancomycin-imprinted silica NPs; (**d**) vancomycin with non-imprinted silica NPs; (**e**) trypsin with trypsin-imprinted silica NPs; (**f**) trypsin with non-imprinted silica NPs. All experiments were performed in 50 mM sodium-phosphate buffer, pH 7.0. Error bars represent ±1 standard deviation and are for experiments performed in triplicate.

**Figure 4:**
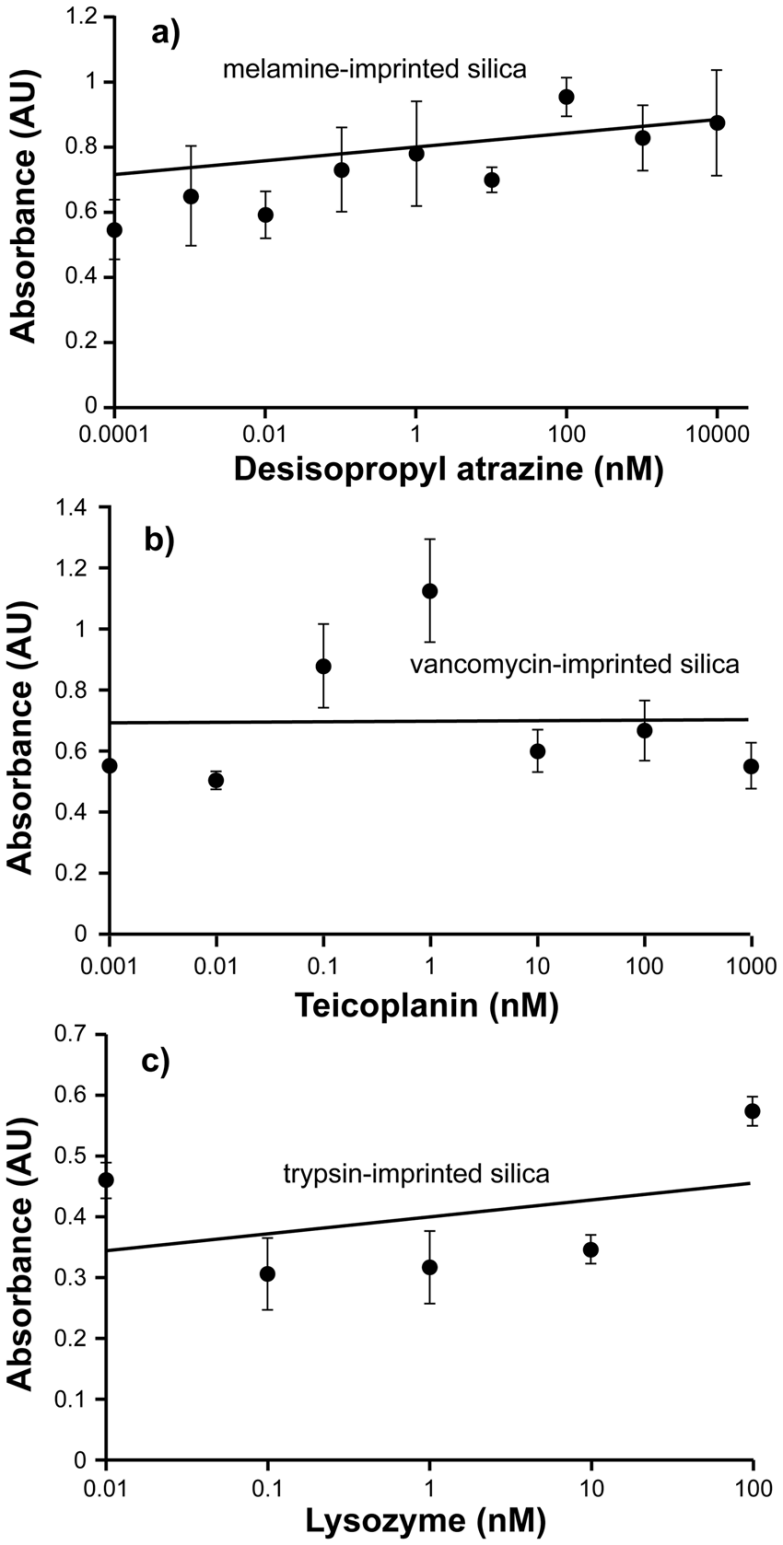
Cross-reactivity assays. Competitive ELISA between free analyte and HRP conjugates corresponding to the templated species, using immobilised silica nanoparticles in 96 well polystyrene microplates. (**a**) Desisopropyl atrazine with melamine-imprinted silica NPs; (**b**) Teicoplanin with vancomycin-imprinted NPs (**c**) Lysozyme with trypsin-imprinted silica NPs. All experiments were performed in 50 mM sodium phosphate buffer, pH 7.0. Error bars represent ±1 standard deviation and are for experiments performed in triplicate.

To analyse the binding properties of silica nanoparticles, ELISA-like assays were performed in a competitive format^11^. This format allows the binding of ligands to immobilised nanoparticles (NPs) to be observed through enzyme-linked signal amplification. It is worth mentioning that the assays reported here were used purely to evaluate and compare the recognition properties of the imprinted NPs. The work presented here was not intended as an example of assay development. Imprinted and non-imprinted nanoparticles were immobilised onto the surface of microplate wells (physically adsorbed) by evaporation of a solution of nanoparticles, forming a visible film (when dry) at the well surface. If undisturbed mechanically, the films are stable and can withstand the assay conditions. During the assay there is competition for binding between free target molecule (the template) and target molecule-horseradish peroxidase (HRP) conjugate to the imprinted film^11^. In this format, increasing concentrations of free target result in a decrease in the signal (absorbance at 450 nm) showing the specific nature of the imprinted binding sites. Assays were performed for the three templates, melamine, vancomycin and trypsin (Fig. 3a to f). To prove the imprinting effect, non-imprinted NPs were used as control. In control assays there was no specific response proportional to the concentration of melamine (Fig. 3b) or vancomycin (Fig. 3d), with a small response (~7%, Fig. 3f) of that observed for the trypsin-imprinted NPs. It is also clear that the melamine assay has an almost negligible response (low sensitivity) when compared with the assays performed with higher molecular weight targets. This can be attributed to possible steric hindrance from the melamine/HRP conjugate and/or to the limited type of functionalities present in the silica, leading to lower numbers of high-affinity imprints when it comes to binding/recognition of small molecules (with few functional groups), such as melamine. The dissociation constant for the melamine imprinted silica NPs binding to melamine immobilised on gold was determined to be 1.2 × 10^−6^ M in experiments carried out on a Biacore 3000 SPR platform (Supplementary data Fig. S1). Larger templates, which can mitigate steric issues, also possess a greater number of functional groups that can establish interactions with the silica are probably more appropriate targets, as exemplified here for vancomycin and trypsin. Cross-reactivity assays were carried out with molecules bearing moieties closely related with those of the templates used during imprinting. This included desisopropyl atrazine (a substituted triazine) with melamine-imprinted NPs and teicoplanin (a glycopeptide antibiotic) with vancomycin-imprinted NPs. For trypsin-imprinted NPs, a protein with a similar isoelectric point (lysozyme) was used. Competitive ELISA assays were performed as described in the Experimental Section, using the analogues mentioned above in place of the imprinted target, but with the HRP-conjugate appropriate for the imprinted NPs. In all cases, no concentration dependent response was observed (Fig. 4a to c).

**Figure 5:**
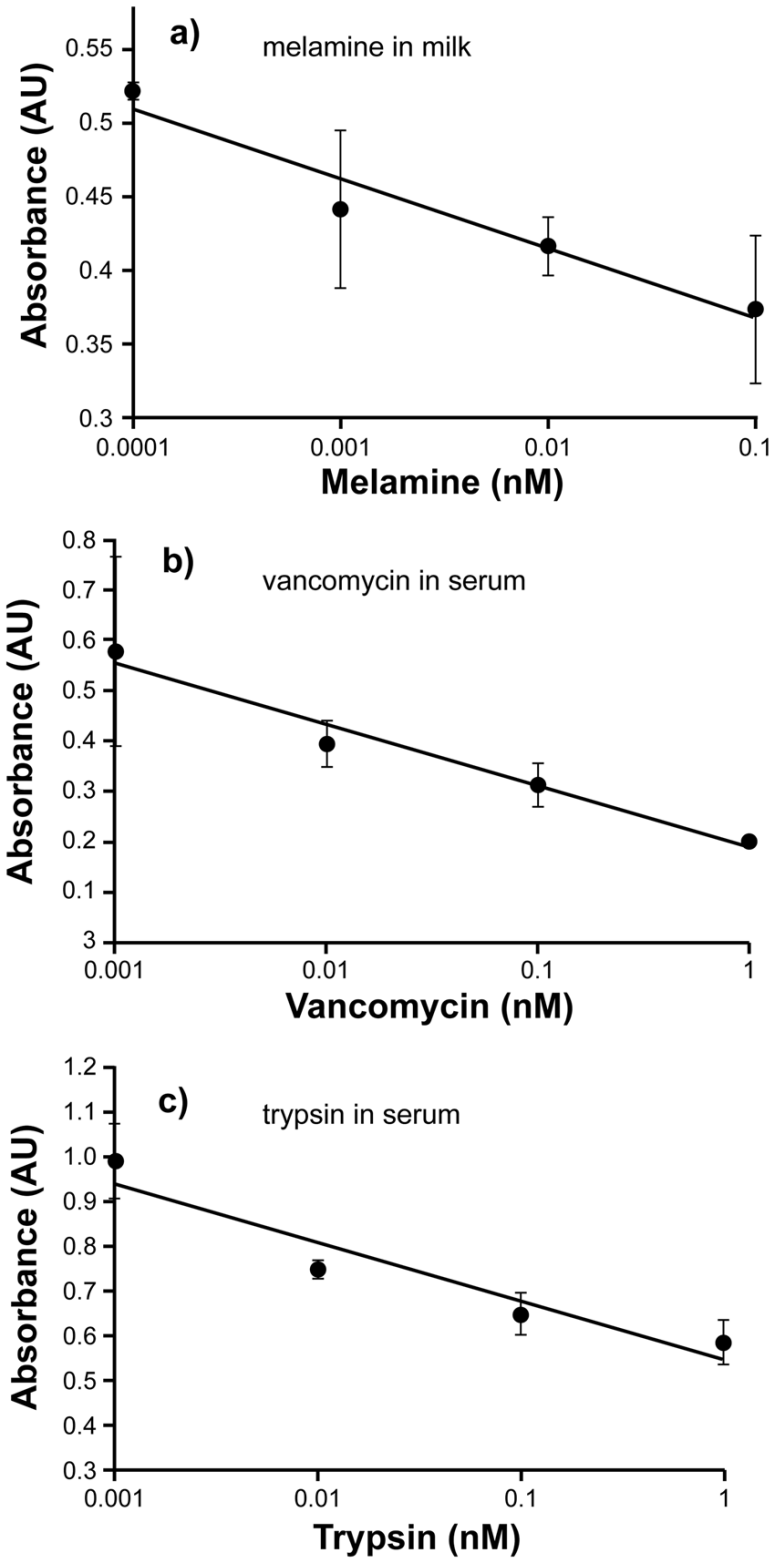
Assays in complex sample matrices spiked with the respective templates. Competitive binding between free analyte and corresponding HRP conjugates in ELISA format using immobilised silica nanoparticles in 96 well polystyrene microplates. In all cases the linear ranges are depicted, no response was observed for lower analyte concentrations whilst saturation is observed at higher concentrations. (**a**) Melamine binding to melamine-imprinted silica NPs in milk; (**b**) vancomycin binding to vancomycin-imprinted NPs in blood serum; (**c**) trypsin binding to trypsin-imprinted silica NPs in blood serum. Melamine experiments was performed in 0.5% v/v milk solution and vancomycin and trypsin in 2.5% v/v blood serum solution, in all cases diluted in 50 mM sodium-phosphate buffer, pH 7.0. Error bars represent ±1 standard deviation and are for experiments performed in triplicate.

Further tests were performed in complex media to assess the performance and potential suitability of the imprinted NPs for analysis in real samples. To this end, melamine-imprinted NPs were tested in spiked cows’ milk; vancomycin and trypsin-imprinted NPs in spiked blood serum. Assays were performed as described below. Although an evident reduction in the linear ranges of the assays was noticed (together with an increased standard deviation for the melamine assay), each of the imprinted NPs was still capable of recognising its respective target in the complex sample matrices (Fig. 5a–c). Due to the low saturation limit of the assays (ranging from 0.1 to 1 nM), and the concentrations of both analytes found in real samples (up to 5 mM melamine in milk^29^ and up to 35 μM for vancomycin in blood serum^30, 31^), spiked samples were diluted in phosphate buffer before analysis. This is advantageous as it helps reduce matrix interferences whilst still allowing for low detection limits^11^. Trypsin was also measured in serum, although this enzyme is not present in blood, the rationale being to assess the performance of the imprinted NPs in a complex mixture which includes various other proteins.

## Materials and Methods

Materials and other methods are described in the Supplementary Material.

### Solid-Phase Imprinting

Silica nanoparticles (50 mg) of 12 nm diameter were suspended in 50 mM phosphate buffer, pH 7.2, (50 mL) and mixed with beads bearing the immobilised template (60 g). Incubation was performed for 1–6 hours, after which the mixture was decanted into a fritted 60 mL solid phase extraction cartridge and washed with distilled water at room temperature (8 bed volumes). The imprinted nanoparticles were then collected by warming the cartridge to 70 °C and eluting the solid phase with water at 70 °C (4 × 20 mL). The yield was determined gravimetrically following evaporation of an aliquot of the solution collected after the hot elution step.

### Immobilisation of Nanoparticles on the Microplate Surface and Performance of the Assay

Aliquots of a suspension (1 mg mL^−1^) of imprinted nanoparticles (40 μL) were dispensed into the wells of a 96 well microplate and the solution allowed to dry overnight at room temperature. Each well was washed with PBS, pH 6.5, (2 × 250 μL) before filling with 300 μL of blocking solution (1% BSA and 1% Tween-20) and allowing to stand for 1 hour. Blocked wells were washed with PBS, pH 6.5, (2 × 250 μL). Conjugate solution (3 μL) was mixed with solutions of free analyte (2.5 mL), varying in concentration from 10^−12^ to 10^−3^ M. Aliquots (100 μL) of these solutions were dispensed into wells of the microplate. After incubation for 1 hour at room temperature, wells were washed with blocking solution, (300 μL, see above for composition). Finally TMB solution (100 μL) was added to each well and incubated for 10–30 min at room temperature. The reaction was stopped by the addition of 0.5 M H_2_SO_4_ (100 μL per well). The optical absorbance at 450 nm was measured using a microplate reader (Dynex Technologies Inc., UK). In the assays with real sample matrices, melamine was diluted in 0.5% milk solution in phosphate buffer pH 7.0 and the vancomycin and trypsin in 2.5% donkey serum solution in phosphate buffer pH 7.0.

## Conclusions

In summary, we have introduced and demonstrated the feasibility of a new, simple and efficient method to prepare molecularly imprinted (biomimetic) silica nanoparticles using commercial silica as a seed material. The imprinting process is based on the Ostwald ripening of silica nanoparticles in the presence of templates immobilised on a solid support. The ripening process is performed under physiological conditions, so in addition to being environmentally benign, it should be generally appropriate for the imprinting of biomolecules in a conformation close to their natural state. Exposure of the imprinted material to the ripening conditions in the absence of templates results in “erasure” of the template memory, allowing the material to be recycled. Molecular imprinting was successfully performed on templates with molar masses, ranging from melamine (126.12 g mol^−1^), through the glycopeptide vancomycin (1449.3 g mol^−1^) to the protein trypsin (23.3 kDa). The imprinting effect and performance of the prepared silica nanoparticles was assessed by competitive (ELISA-like) assays where the imprinted silica replaced the antibodies used in the traditional immunoassay. The assays demonstrated a clear effect of the imprinting step on the molecular recognition capability of the silica. Preferential binding of the target (template) molecule was observed over related analogues, whereas no molecular recognition properties were apparent with non-imprinted silica. In addition, the imprinted nanoparticles were capable of selectively binding to their respective targets even in complex sample matrices such as milk and blood serum, although a reduction in the linear range of the assays was noted. Due to ease of preparation and good molecular recognition performance of the synthesised materials, molecular imprinting via Ostwald ripening can be considered a suitable approach, as an alternative to polycondensation, for the preparation of affinity sol-gel nanoparticles. Like other imprinted materials, these could find applications in assays, sensors and in affinity separations.

## Electronic supplementary material

Supplementary information
