# Supplementary material for: Biomimetic Silica Nanoparticles Prepared by a Combination of Solid-Phase Imprinting and Ostwald Ripening
Source: Sci Rep. 2017 Sep 14;7:11537. doi: 10.1038/s41598-017-12007-0 (PMC5599519; doi:10.1038/s41598-017-12007-0)
Supplement: Supplementary file 1 — Supplementary information [file 41598_2017_12007_MOESM1_ESM.pdf]

Biomimetic Silica Nanoparticles Prepared by a Combination of Solid-Phase Imprinting and Ostwald Ripening

Elena Piletska,[a*] Heersh Yawer,[a] Francesco Canfarotta,[b] Ewa Moczko,[c] Katarzyna Smolinska-Kempisty,[a] Stanislav S. Piletsky,[a] Antonio Guerreiro,[a] Michael J. Whitcombe,[a*] and Sergey A. Piletsky[a]

*ep219@le.ac.uk; mw319@le.ac.uk

[a]Department of Chemistry, College of Science and Engineering, University of Leicester, LE1 7RH, UK

[b]MIP Diagnostics Ltd., Fielding Johnson Building, University of Leicester, LE1 7RH, UK.

[c]Current address: Universidad Católica de la Santísima Concepción, Facultad de Ciencias, Departamento de Química Ambiental, Alonso de Ribera 2850, Concepción, Chile.

Supplementary Material

Materials 2

Activation of the Solid Phase and Immobilisation of Templates 2

Dynamic Light Scattering (DLS) Analysis 3

Preparation of template-HRP conjugates 3

- **Conjugation of melamine and vancomycin:** 3
- **Conjugation of trypsin:** 3

Surface Plasmon Resonance characterisation of binding to melamine 4

Materials

Trypsin, vancomycin, melamine, desisopropyl atrazine, lysozyme, N-[3-(trimethoxysilyl)propyl]ethylenediamine (TMSPED), sodium di-hydrogen phosphate monohydrate, di-sodium hydrogen phosphate, sodium hydroxide, hydrochloric acid, glutaraldehyde, ethanolamine hydrochloride, bovine serum albumin (BSA), horseradish peroxidase (HRP), tween-20, 2-morpholinoethanesulfonic acid (MES), *N*-hydroxysuccinamide (NHS), 1-ethyl-3 (3-dimethylaminopropyl)-carbodiimide hydrochloride (EDC), 3,3′,5,5′-tetramethylbenzidine (TMB), methanol, ethanol, toluene, acetone, donkey serum and silica nanoparticles (12 nm diameter) were from Sigma Aldrich, UK. Teicoplanin was from Fisher Scientific. Fresh skimmed milk was purchased from Tesco, UK. Nunclon 96 well microplates (polystyrene) were from Thermo Scientific, UK. Ultra 30 and 50 kDa MWC centrifugal dialysis filter units were from Amicon UK. Glass beads (Spheriglass® 2429 CP00, 53-106 μm diameter), were from Blagden Chemicals.

Activation of the Solid Phase and Immobilisation of Templates

All solution incubation steps involving glass beads were performed with 0.4 g beads per mL of solution. Glass beads were boiled in 1M NaOH for 10 min and washed extensively with distilled water until the pH of the beads slurry was ~7.5. Beads were washed with acetone, drained and left to dry at 80 °C. Dry beads were placed in dry toluene and incubated overnight with 2 % v/v TMSPED at room temperature. The silanised beads were washed with 5 volumes of acetone and three volumes of ethanol before drying on a sintered-glass funnel. Beads were then incubated with 5 % v/v glutaraldehyde in 10 mM PBS buffer, pH 7.4, for two hours, then washed with 8 volumes of water on a sintered-glass funnel. Afterwards, glutaraldehyde-activated beads were used immediately for surface immobilisation of the template. This was achieved by incubating the beads with a solution of either melamine, vancomycin or trypsin (5 mg mL^{-1}) in PBS, pH 7.2, overnight at 4 °C. In the case of melamine, NMP (10% v/v) was also added as co-solvent. Finally, the glass beads were washed with water and dried under vacuum, then stored at 4 °C until used.

Dynamic Light Scattering (DLS) Analysis

The kinetics of evolution of the size of silica nanoparticles (1 mg mL^{-1}) in phosphate buffer (50 mM, pH 7.2) was monitored by DLS. All DLS measurements were performed at room temperature (25 ± 2 °C), with a Zetasizer NanoZS (Malvern Instruments).

Preparation of template-HRP conjugates

HRP (20 mg) was dissolved in 0.1 M MES buffer, pH 6.0 (20 mL) and mixed with EDC (0.8 mg) and NHS (1.2 mg). The mixture was allowed to stand for 15 minutes before washing on a 30 kDa Amicon centrifugation filter.

Conjugation of melamine and vancomycin: Aliquots (20 mL, 1 mg mL^{-1}) of either molecule in PBS were immediately added to the washed and activated HRP followed by incubation for 2 hours. After conjugation, the template-HRP conjugate was washed on a 30 kDa centrifugation filter with water (10 × 10 mL).

Conjugation of trypsin: Trypsin (1 mg mL^{-1} in 20 mL PBS) was immediately added to the washed and activated HRP then incubated for 2 hours. Ethanolamine hydrochloride was then added to the HRP-trypsin solution to a final concentration of 1 mM and incubated a further 15 min. The conjugate solution was washed with water (10 × 10 mL) on a 50 kDa centrifugation filter. After washing, the conjugate was dissolved in 2 mL of distilled water and stored at –18 °C.

Biacore experiments

The interactions of melamine-specific silica nanoparticles with specific surfaces have been tested using Biacore 3000 instrument (GE Healthcare Life Sciences, USA) at 25 °C using PBS **(**0.01 M phosphate buffer, 0.0027 M potassium chloride and 0.137 M sodium chloride, pH 7.4) as a running buffer at a flow rate of 15 μL min^{-1}. The suspensions of melamine-specific silica nanoparticles in water were filtered through the syringe filters with pore diameter 0.45 μm (Supelco, UK) and diluted in PBS for the analysis first 10 times, then in 2 × increments. Silica nanoparticles were imprinted by Ostwald ripening in the presence of immobilised melamine for 2 h. The dissociation constant (K_D) was calculated from plots of the equilibrium biosensor response (Fig. S1) as a function of nanoparticles concentration using the BIA-evaluation 3.1 software. The calculated K_D for silica NP imprinted for 2 h was estimated to be 1.2×10^{-6} M.

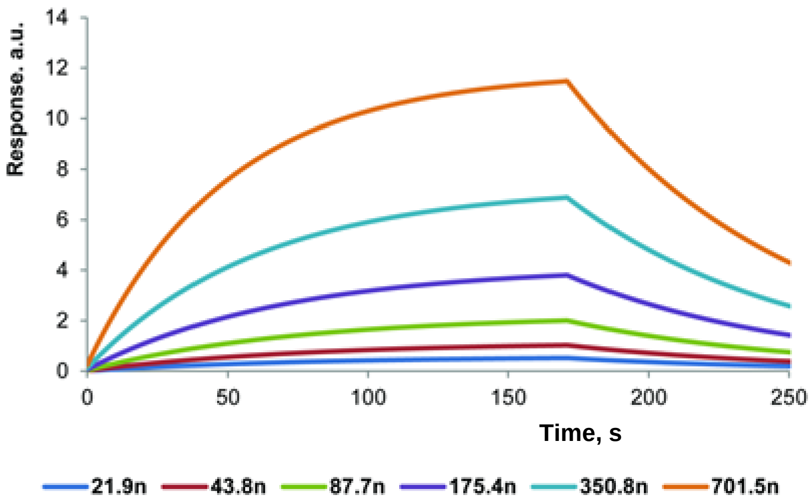

Figure S1. Sensorgram of time-dependent binding of melamine-specific silica NPs to melamine-coated surface of Biacore sensor chip. The molar concentrations (from 21.9 to 701.5 nM) of the injected particles are shown in the key below the graph.
